# Age-Related Changes of Myelin Basic Protein in Mouse and Human Auditory Nerve

**DOI:** 10.1371/journal.pone.0034500

**Published:** 2012-04-05

**Authors:** Yazhi Xing, Devadoss J. Samuvel, Shawn M. Stevens, Judy R. Dubno, Bradley A. Schulte, Hainan Lang

**Affiliations:** 1 Department of Pathology and Laboratory Medicine, Medical University of South Carolina, Charleston, South Carolina, United States of America; 2 Department of Otolaryngology – Head & Neck Surgery, Medical University of South Carolina, Charleston, South Carolina, United States of America; University of Washington, Institute for Stem Cells and Regenerative Medicine, United States of America

## Abstract

Age-related hearing loss (presbyacusis) is the most common type of hearing impairment. One of the most consistent pathological changes seen in presbyacusis is the loss of spiral ganglion neurons (SGNs). Defining the cellular and molecular basis of SGN degeneration in the human inner ear is critical to gaining a better understanding of the pathophysiology of presbyacusis. However, information on age-related cellular and molecular alterations in the human spiral ganglion remains scant, owing to the very limited availably of human specimens suitable for high resolution morphological and molecular analysis. This study aimed at defining age-related alterations in the auditory nerve in human temporal bones and determining if immunostaining for myelin basic protein (MBP) can be used as an alternative approach to electron microscopy for evaluating myelin degeneration. For comparative purposes, we evaluated ultrastructural alternations and changes in MBP immunostaining in aging CBA/CaJ mice. We then examined 13 temporal bones from 10 human donors, including 4 adults aged 38–46 years (middle-aged group) and 6 adults aged 63–91 years (older group). Similar to the mouse, intense immunostaining of MBP was present throughout the auditory nerve of the middle-aged human donors. Significant declines in MBP immunoreactivity and losses of MBP^+^ auditory nerve fibers were observed in the spiral ganglia of both the older human and aged mouse ears. This study demonstrates that immunostaining for MBP in combination with confocal microscopy provides a sensitive, reliable, and efficient method for assessing alterations of myelin sheaths in the auditory nerve. The results also suggest that myelin degeneration may play a critical role in the SGN loss and the subsequent decline of the auditory nerve function in presbyacusis.

## Introduction

Age-related hearing loss (presbyacusis) affects about half the population over 75 years of age [1]. Studies of temporal bones from older human donors have shown that one of the most common pathological changes seen in age-related hearing loss is the degeneration of spiral ganglion neurons (SGNs) [2]–[5]. Primary degeneration of the auditory nerve has also been demonstrated in animal models and humans through mechanisms not solely related to hair cell loss [6]–[9]. Definition of the cellular and molecular mechanisms underlying human SGN degeneration is an important step toward a better understanding of the pathophysiology of this process and generating improved methods of diagnosis and treatment. However, knowledge of age-related molecular alterations in the human spiral ganglion remains very limited due to the complexity of inner ear structures and the lack of specimens processed specifically for this purpose.

Two populations of SGNs are present in the mammalian ear [10]–[13]. Bipolar type I neurons comprise about 95% of the afferent neurons in the cochlea. Their peripheral processes synapse both directly and indirectly with a single inner hair cell, which in turn constitute the primary sensory receptors in the cochlea. The remaining type II neurons (about 5%) are unmyelinated and innervate multiple outer hair cells but their function is still largely unknown *in vivo* [14]–[16]. Both the peripheral and central processes of type I neurons are enveloped in a thick myelin sheath that serves as an electrical insulating material. The integrity of the compacted, multi-lamellar myelin sheath plays an important role in the proper functioning of neurons, e.g., in determining the speed of neural transmission [17]. As in most mammalian species, the cell bodies of type I SGNs in adult mice are sheathed in myelin. In contrast, a great majority of the somata of human type I SGNs are unmyelinated as demonstrated by several light and electron microscopic studies [18]–[22], suggesting that neural conduction may be slower in the human auditory nerve compared with other mammalian species.

Studies of several animal models have demonstrated that a deficiency of myelin in the spiral ganglion significantly degrades auditory nerve activity [23]–[26]. Age-related progressive degeneration of myelin and the loss the myelinated nerve fibers have been reported in other regions of the nervous system including the cerebral cortex [27], corpus callosum [28], [29], optic nerve [30] and cochlear nucleus [31]. Several models of age-related neurodegenerative disorders, including Alzheimer's disease, also support this finding [29], [30], [32]. Based on these observations, we hypothesized that age-related degeneration of myelin sheaths may occur in the human auditory nerve and that these changes may contribute to the decline of auditory nerve function in presbyacusis.

Electron microscopic observation is the standard procedure used to examine changes in the fine structure of myelin. However, the preservation of myelin ultrastructure is exquisitely sensitive to fixation artifacts, in particular fixation delays [27], [28], which render almost all human temporal bone preparations unsuitable for electron microscopic analysis. In addition, although neuron-Schwann cell contact, axon-Schwann cell contact, and axon engraftment have been documented in numerous animal studies [17], [33], [34], the dynamics of myelin membrane deposition and membrane compaction cannot be captured by electron microscopy. Thus, alternative non-ultrastructural approaches are necessary to assess pathological changes in human auditory nerves associated with presbyacusis and other forms of sensorineural hearing loss.

Myelin basic proteins (MBPs) are the major constituents of the myelin sheath produced by oligodendrocytes and Schwann cells in the nervous system and comprise 30% of the total protein found in myelinating cells [35]. MBP is part of a family of proteins consisting of multiple polypeptide chains ranging from 14 to 21.5 kDa in molecular weight. This protein family is the product of a large gene complex called Golli (Genes of OLigodendrocytes Linage) having 11 exons in mice and 10 exons in human. MBP is essential to the formation of nervous system myelin and has been termed the “executive molecule of myelin” [36]. In the auditory system, MBP mRNA expression can be detected in the mouse auditory nerve on postnatal day 2 and reaches its peak level on postnatal day 10 [37]. A decline of MBP expression has been reported in the auditory nerve of Tmprss1 (a member of the type II transmembrane serine protease gene family) deficient mice [38] and in postnatal rats exposed to toxic levels of carbon monoxide [39]. However, little is known about changes in the expression pattern of MBP in the aging auditory system or the impact of these changes on auditory nerve degeneration and functional declines in presbyacusis.

In this study, we examined age-related degeneration of the myelin sheath in the human auditory nerve using an immunohistochemical assay for MBP. We compared the results in human temporal bones with correlative pathological and functional studies of an aging mouse model to begin to assess the influence of myelin degeneration on declines in auditory nerve function in presbyacusis. The expression pattern of MBP and the density of MBP^+^ fibers were characterized in the auditory nerves using both conventional and confocal microscopy. Our data show that changes in the MBP immunostaining pattern and losses of MBP^+^ nerve fibers are associated with ultrastructural changes in the myelin sheath and a significant decline of auditory nerve function in aged CBA/CaJ mice. The results also demonstrate that a dual immunostaining approach combined with high-resolution confocal microscopy can detect degenerative changes in the myelin sheath prior to the loss of auditory nerve fibers.

In the human temporal bones, intense MBP^+^ staining was seen in the auditory nerve both peripherally within the osseous spiral lamina and Rosenthal's canal as well as in the glial transition zone of the auditory nerves. MBP immunostaining patterns and the number of MBP^+^ nerve fibers were compared between middle-aged and older human temporal bones. Similar to the mouse model, a reduction in the intensity of MBP immunoreactivity and a significant loss of MBP^+^ auditory nerve fibers was observed in the inner ears of older humans.

## Results

### Age-related hearing loss and decline of auditory nerve function in CBA/CaJ mice

**Figure 1:**
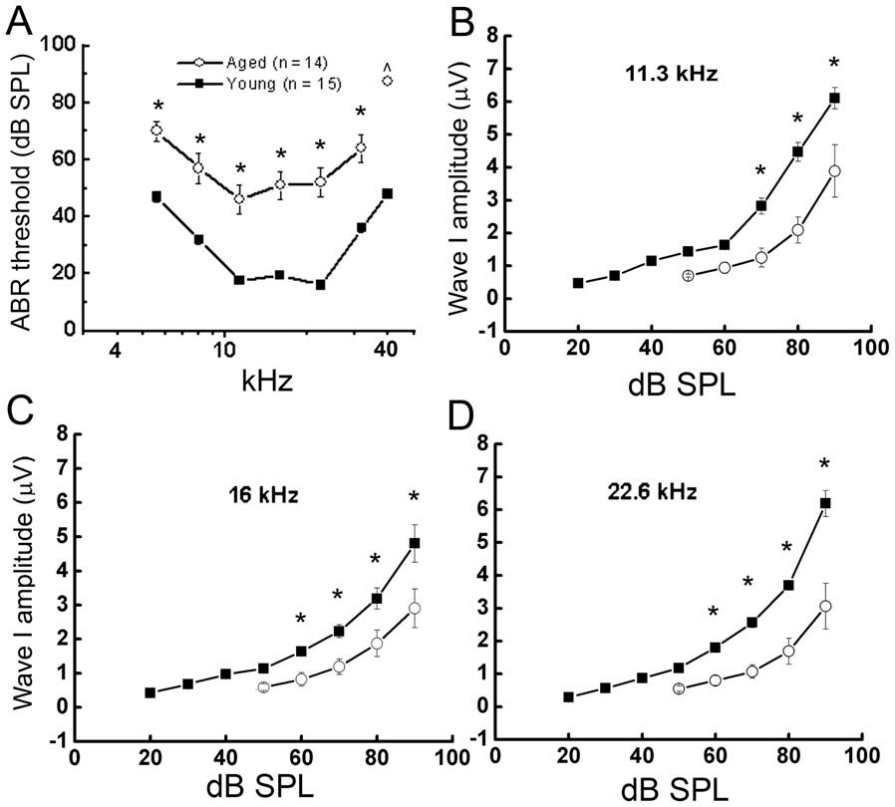
Decline of auditory nerve function in aged CBA/CaJ mice. **A:** Comparisons of ABR thresholds for wave I obtained from 1–3 month-old and 23–27 month-old mice. It showed significant ABR threshold shifts in older mice across all frequencies tested (5.6, 8, 11.3, 16, 22.6, 32, and 40 kHz). No ABR response was detected at 40 kHz in older mice. **B, C, D:** Mean wave I amplitude vs. level functions. The data show increased ABR thresholds and decreased wave I amplitudes in aged mice at 11.3, 16, and 22.6 kHz. All data are presented as mean ± SEM. Asterisks indicated statistically significant differences at the indicated frequencies (*p*<0.05).

CBA/CaJ mice were originally bred for longevity research and the incidence of spontaneous tumor formation [40]. CBA/CaJ is one of two CBA inbred strains (another strain is CBA/J) that is widely used as a “good hearing” standard or “normal aging” model in hearing research [41]–[48]. Comparisons of the mean ABR thresholds for 1–3-month-old and 23–27-month-old CBA/CaJ are shown in Figure 1A. Significant threshold shifts occurred in the aged mice at all frequencies tested compared to those in the young-adult controls (unpaired t-test, *p*<0.05).

We also employed ABR wave I amplitude input/output (I/O) functions to assess the gross activity of the mouse auditory nerve. As shown in Figures 1B–D, the maximum wave I amplitudes and slopes of the I/O functions in the aged group were reduced significantly at 11.3, 16, and 22.6 kHz, indicating a significant reduction in ABR wave I suprathreshold amplitudes in aged mouse ears (n = 15) compared to those of young adult controls (n = 14) (*p*<0.05).

### Ultrastructural changes in the myelin sheath in aged CBA/CaJ mice

**Figure 2:**
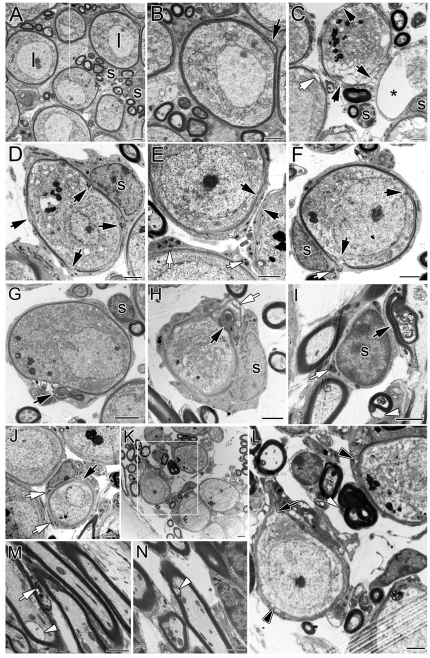
Age-related ultrastructural changes in CBA/CaJ mouse spiral ganglion. **A, B:** Normal type I (I) SGNs and myelinating Schwann cells (S) in the middle turn of a young adult mouse. **B** shows a higher magnification view of the boxed area in A. Chromatin is evenly distributed in the prominent nucleus and the neuronal perikaryon is surrounded by a myelin sheath and by the peripheral process of a Schwann cell. The majority of myelin sheaths enclosing type I SGNs are tightly laminated with no discontinuities. Although a separation of myelin sheath layers enveloping SGNs was occasionally seen in young adult mice (arrow), no discontinuities in the myelin sheath were seen in the young mice. **C:** A section from the basal turn of a 27-month-old mouse showing SGNs in various stage of degeneration. **A** degenerating SGN surrounded by a thinned and broken myelin sheath (black arrows) and numerous electron dense inclusions are present in the cytoplasm. On the left, a gap (white arrow) is present in the myelin sheath enclosing a relatively healthy appearing SGN. On the right, debris from a dead SGN is enclosed by a myelin sheath and a Schwann cell process (asterisk). **D, E, F:** Abnormalities of the myelin sheath in the middle turn of another 27-month-old mouse. Black arrows indicate areas of broken (**D, F**) and loose (**E**) myelin lamellae enclosing both degenerative and normal SGNs. The dense inclusions in the cytoplasm of Schwann cells are indicated by white arrows (**E**). **G, H, I:** Degenerative changes in the middle turn of a 27-month-old mouse. Abnormal partially collapsed or folded myelin sheaths (black arrows) and balloon-like features (white arrowhead) are seen surrounding the perikarya of SGNs (**G**, **H**) and their processes (**I**). **J:** Numerous electron dense inclusions in the cytoplasm of a Schwann cell in the center (white arrows). The Schwann cell associated SGN is surrounded by a broken myelin sheath (black arrow). **K, L, M, N:** Abnormal collapsed and folded myelin sheaths (white arrow), broken myelin sheaths (black arrow), balloon-like features (white arrowhead), and vacuole-like spaces (black arrowheads) surrounding the perikarya of SGNs (**J,L**) and auditory nerve processes within RC (**L**) and OSL (**M,N**). **L** is a higher magnification view of the boxed area in **K**. A white arrow in **M** points electron dense inclusions in the cytoplasm of a Schwann cell. All images in **J–N** were taken from a 28-month-old mouse. Scale bars = 2 µm in **A–N**.

The most common neuronal cell type in the spiral ganglion of the adult mouse is the type I (myelinated) SGN. The unique ultrastructural features of type I SGNs and their processes from a young adult mouse are shown in figures 2A and 2B. The type I neuron is characterized by: 1) a perikaryon completely enveloped by a thick, compact myelin sheath; 2) numerous cytoplasmic organelles that give the SGN a granular and relatively dark appearance; and 3) a round to ovoid nucleus with evenly distributed chromatin (euchromatin) that appears less electron dense. Myelin sheaths enclosing the neural processes are thicker than those enveloping the perikarya. In young mice, the myelin sheaths enveloping the perikarya are intact and tightly laminated without any discontinuities, although a slight separation of myelin sheath layers appears in a few SGNs (Fig. 2B).

In the 23–28-month-old mouse ears (n = 4), myelin sheaths surrounding SGNs (Figs. 2D–H, J–L) and their nerve processes (Figs. 2I, M, N, L) exhibited several forms of degeneration. Disorganization of the myelin sheath was observed in the majority of SGNs in all three turns from four aged mice. Specific features of the degenerating myelin sheath included thinner lamellae (Fig. 2C), loose lamellae (Fig. 2E), split myelin lamellae with discontinuities (Figs. 2D, F, J), and vacuole-like inclusions in the cytoplasm of Schwann cells (Fig. 2L). Disorganization of myelin was seen both in SNGs with severe degenerative changes (Figs. 2C, D, L) and in SGNs with a relatively normal appearance (Figs. 2E, F, G, J).

Disrupted myelin sheaths also were seen in auditory nerve processes within Rosenthal's canal (Fig. 2L) and the osseous spiral lamina (Figs. 2I, M, N). These degenerative myelin sheaths were characterized by folded or collapsed lamella (Figs. 2I, L) and balloon-like features (Fig. 2I). In addition, dense cytoplasmic inclusions were seen in the myelin-forming Schwann cells in all three turns of the aged mouse ears (Figs. 2E, F, H, J). Similar dense inclusions were not seen in the young adult mice (Figs. 2A, B).

The mean thickness of the compact myelin sheath in young adult mice as measured on electron micrographs was 0.32±0.02 µm in the peripheral processes and 0.11±0.01 µm in the sheath enveloping SGN cell bodies within Rosenthal's canal. Thicknesses were 0.41±0.03 µm in the peripheral processes and 0.12±0.01 µm around the SGN cell bodies within Rosenthal's canal in aged mice. No age-related significant difference was seen in the thickness of compact myelin sheaths between the two age groups (unpaired t-test, *p*>0.05).

### Age-related changes in myelin basic protein immunoreactivity in mouse auditory nerves

**Figure 3:**
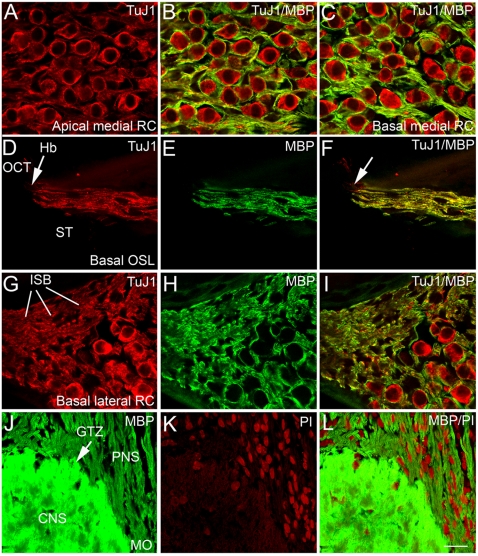
Immunohistochemical localization of myelin basic protein (MBP) in young adult CBA/CaJ mouse ears. **A, B, C:** Confocal images of SGNs labeled with anti-class III β-tubulin (TuJ1, red) and anti-MBP (green) within the medial portion of Rosenthal's canal (RC) in the apical (**A,B**) and basal (**C**) turns of a one-month-old mouse. TuJ1 is a neuronal marker that preferentially labels the cytoplasm of type I SGNs and their processes. MBP labeling of myelin sheaths revealed a honeycomb-like staining pattern in the spiral ganglion. **D, E, F:** Myelinated peripheral processes in the osseous spiral lamina (OSL) of the basal turn of a three-month-old mouse stained strongly for MBP. A white arrow points to the habenula perforata (Hb) where auditory fibers lose their myelinated sheaths. **G, H, I:** MBP^+^ fibers in the lateral portion of RC in the basal turn of a three-month-old mouse. MBP^+^ myelinated fibers and MBP^−^ unmyelinated fibers form intraganglionic spiral bundles. **J,K,L:** MBP^+^ fibers in the glial transition zone (GTZ) of the modiolus (MO) ensheathed by oligodendrocytes (CNS) and Schwann cells (PNS). Nuclei were counterstained with PI (red). Organ of Corti, OCT; Scala tympani, ST. Scale bars, 12 µm in **L** (applies to **A–L**).

Myelin basic protein (MBP) is one of three major myelin proteins (P0, PMP22, MBP) in the peripheral nervous system [49]. The aforementioned finding of pathological changes in the myelin sheaths of the aged mice led us to investigate the MBP expression pattern in both young adult and aged mouse ears. In young adult CBA/CaJ mice, high-resolution confocal images revealed intense immunostaining for MBP surrounding SGNs and their processes (Fig. 3). Class III β-tubulin (TuJ1) stained the microtubule components of type I SGNs. Most TuJ1^+^ SGNs were enveloped by a MBP^+^ myelin sheath, forming a honey-comb-like staining pattern (Figs. 3A–C). Intense MBP staining was also seen in both the peripheral and central myelinated processes of the auditory nerve (Figs. 3D–L). Intraganglionic spiral bundles in the lateral region of Rosenthal's canal, consist of both myelinated and unmyelinated auditory nerve fibers as previously described [50]. Dual-immunostaining with TuJ1 and MBP revealed myelinated fibers within the intraganglionic spiral bundles (Figs. 2G–I), whereas the unmyelinated fibers were difficult to differentiate.

**Figure 4:**
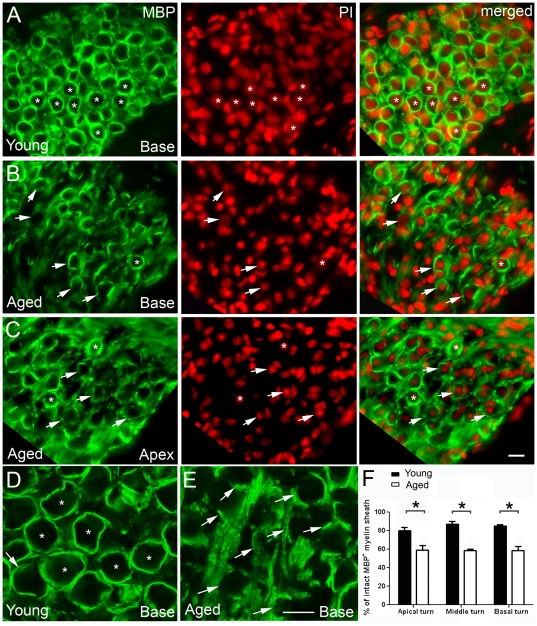
Age-related changes in MBP expression in the CBA/CaJ mouse spiral ganglion. **A:** MBP^+^ myelin sheaths (green) enclose type I SGNs in Rosenthal's canal of the basal turn of a one month-old mouse. Intact MBP^+^ myelin sheaths (asterisks) are seen enveloping most neurons. Propidium iodide (PI) nuclear counterstaining (red) reveals nuclear profiles of both SGNs and Schwann cells. SGNs are identifiable by their larger, more weakly stained spherical nuclei whereas the nuclei of Schwann cells are recognized by their irregular shape and more intense staining. **B, C:** Abnormalities in MBP expression patterns in Rosenthal's canal of aged mice. Numerous SGNs are enclosed only partially by a MBP^+^ myelin sheath (arrows) and some SGNs appear to lose their MBP^+^ myelin sheath. Asterisks indicate two SGNs with normal-looking MBP^+^ myelin sheaths. **D, E:** Confocal images of myelin sheaths enveloping neurons from young (**D**) and aged (**E**) mice. Intact MBP^+^ myelin sheaths are indicated by asterisks while broken MBP^+^ myelin sheaths are indicated by white arrows. **F:** Quantitative analysis of SGNs with intact MBP^+^ myelin sheaths in young and aged mouse ears. The MBP^+^ myelin sheath was considered intact if it enclosed more than 80% of the outline of the perikarya. A significant decline in the percentage of intact MBP^+^ myelin sheath was found in all three cochlear turns in the aged mouse ears compared to young controls (n = 5–7 per group; *P*<0.01; asterisk). Scale bar, 10 µm in **C** (applies to **A–C**); 10 µm in **E** (applies to **D–E**).

**Figure 5:**
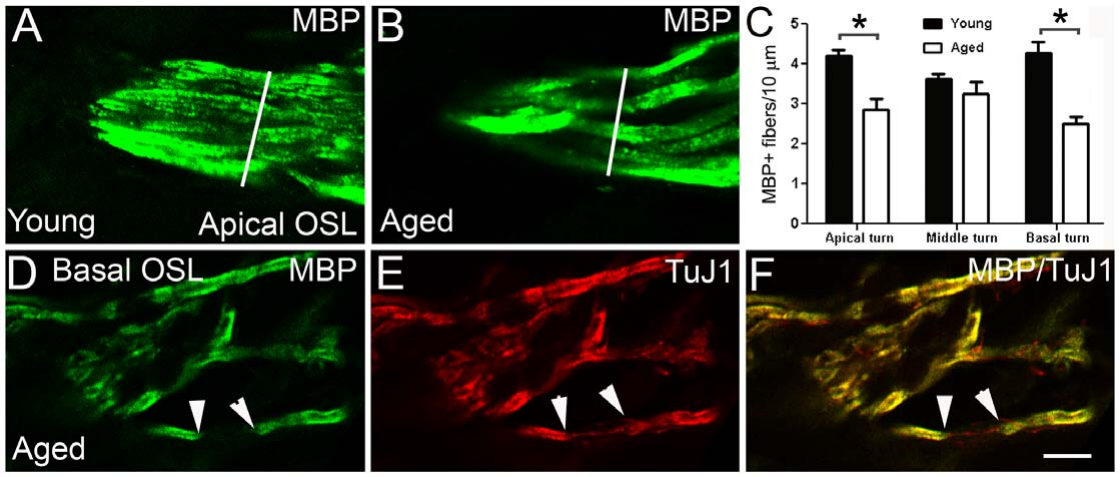
Age-related degeneration of MBP^+^ nerve fibers in aged CBA/CaJ mice. **A, B:** Confocal images of the MBP^+^ nerve fibers in the osseous spiral lamina of a one-month-old (**A**) and a 26-month-old (**B**) mouse. White lines indicate the areas where MBP^+^ fibers were counted (please see Materials and Methods for detailed information on MBP^+^ fiber counting). **C:** Mean density of MBP^+^ fibers within osseous spiral lamina of the apical, middle, and basal turns from young and aged mouse ears (n = 3–5 per group). A significant age-related reduction of MBP^+^ fiber density was found within the apical and basal turn (*P*<0.05; asterisk). **D, E, F:** Degeneration of MBP^+^ myelin in the auditory nerve of a 26-month-old mouse. Dual labeling with anti-MBP (green) and anti-class III β-tubulin (TuJ1, red) indicates that partial demyelination (white arrowheads) occurs in the nerve fibers within the OSL. Scale bar, 8 µm in F (applies to **A–F**).

In the aged mouse ear, MBP was expressed in the same locations as in young adult mice (Figs. 4, 5). However, abnormalities in the staining pattern for MBP were seen in the SGNs of old mice. In young adult ears, the MBP^+^ myelin sheath was intact and enclosed the entire SGN (Figs. 4A, D). In contrast, numerous disruptions were present in the MBP^+^ sheath surrounding aged SGNs in all cochlear turns (Figs. 4B, C, E). The MBP^+^ myelin sheaths in many neurons were discontinuous and in some cases appeared to be missing altogether. We completed a quantitative analysis of this myelin disruption by counting the total number of neurons observed within Rosenthal's canal of both young adult and aged ears (in all three turns) and then calculated the percentage of SGNs having an intact MBP^+^ myelin sheath (Fig. 4F). An “intact” myelin sheath was characterized as MBP^+^ sheath enclosing >80% of the SGNs without any disruptions. The data revealed a lower percentage of intact MBP^+^ myelin sheath in the aged mice compared to young adult mice (unpaired t-test, *p*<0.05).

The loss of SGNs and auditory nerve fibers has been reported in aged gerbils [8], [51], rats [52], [53], and in several strains of aging mice [44], [46], [47]. We determined the density of MBP^+^ myelin fibers within the osseous spiral lamina to compare our results with these prior findings. As shown in Figs. 5A–C, the density of MBP^+^ myelinated fibers was significantly reduced in the aged mouse group compared to that of young adult mice (unpaired t-test, *P*<0.05). In addition, confocal images of dual-immunostaining for MBP and TuJ1 revealed that partial demyelination occurs in some nerve processes within the osseous spiral lamina (Figs. 5D–E). We also measured the thicknesses of MBP^+^ myelin sheaths around SGNs on mid-modiolar frozen sections. The mean thicknesses of the MBP^+^ sheaths was 0.55±0.02 µm and 0.49±0.06 µm in the middle and basal turns of young adult mice, respectively, and 0.49±0.04 µm and 0.52±0.02 µm in the middle and basal turns of aged mice, respectively. No significant difference in myelin sheath thickness was found between the two groups (unpaired t-test, *p*>0.05).

### Expression pattern of myelin basic protein in the human auditory nerve

**Figure 6:**
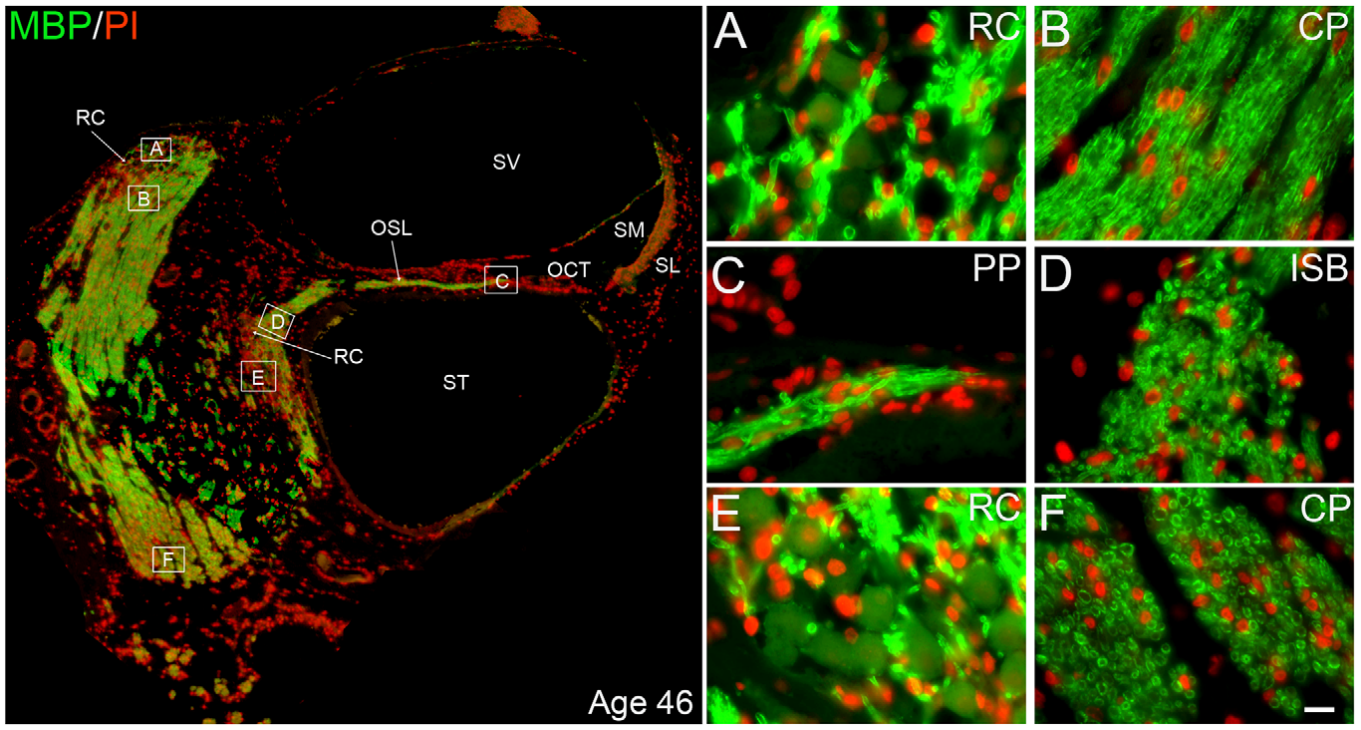
Immunolocalization of MBP in middle-aged human ears. **Left panel:** Low-power view of apical and middle turns from a 46-year-old donor illustrating auditory nerve fibers in different regions stained positively for MBP (green). Nuclei were counterstained with PI (red). **Right panel:** Enlarged images corresponding to the boxed areas labeled **A–F** in the left panel. **A:** MBP^+^ fibers located within Rosenthal's canal (RC) of the apical turn. **B:** MBP^+^ fibers in the central projection (CP) of the auditory nerve within the modiolus close to the apical turn. **C:** MBP^+^ fibers in the peripheral process (PP) of the auditory nerve within the osseous spiral lamina (OSL). **D:** MBP^+^ intraganglionic spiral bundle (ISB) fibers in the middle turn. **E:** MBP^+^ fibers located within Rosenthal's canal of the middle turn. **F:** Transverse section of MBP^+^ fibers in the CP within the modiolus close to the middle turn. Additional abbreviations: OCT, organ of Corti; SL, spiral ligament; SM, scala media; ST, scala tympani; SV, scala vestibuli. Scale bar, 7 µm in **F** applies to **A–F**.

A strong immunostaining reaction for MBP was present in the auditory nerve of all 13 temporal bones from the 10 human donors examined (Table 1). Note that all immunostaining of the human temporal bones was performed on paraffin sections, while staining for MBP in mouse was performed on frozen sections. Figure 6 illustrates the MBP staining pattern in an ear from a 46-year-old donor (H26). Similar to the pattern in the mouse, intense labeling of MBP was seen in peripheral auditory nerve processes in the osseous spiral lamina, and the intraganglionic spiral bundle, as well as in central processes between the SGNs in Rosenthal's canal and more medial auditory nerve within the modiolus. Robust staining for MBP was seen in both middle-aged and aged human ears. Table 1 also lists the death-to-fixation interval for each human temporal bone used in this study. It is notable that a death-to-fixation interval of <6 hours appeared to provide excellent immunostaining results, at least with all 3 of the antibodies used here.

**Table 1:** Temporal bone donor information and immunoreactivity for MBP antibody.

ID Num	Age group	DonorAge/Gender	Death to fixation interval	MBP Immuno-reactivity
**H1**	older	74, female	6 hours	+++
**H3**	older	91, male	4 hours	++
**H4**	older	69, female	2 hours	+++
**H5**	older	63, male	5 hours	++
**H8**	Middle-age	43, female	1 hour	+++
**H10**	Middle-age	46, male	2 hours	+++
**H11**	older	63, male	3 hours	++
**H18**	Middle-age	38, female	2 hours	++
**H19**	older	67, male	2 hours	+++
**H26**	Middle-age	46, female	3 hours	+++

**Figure 7:**
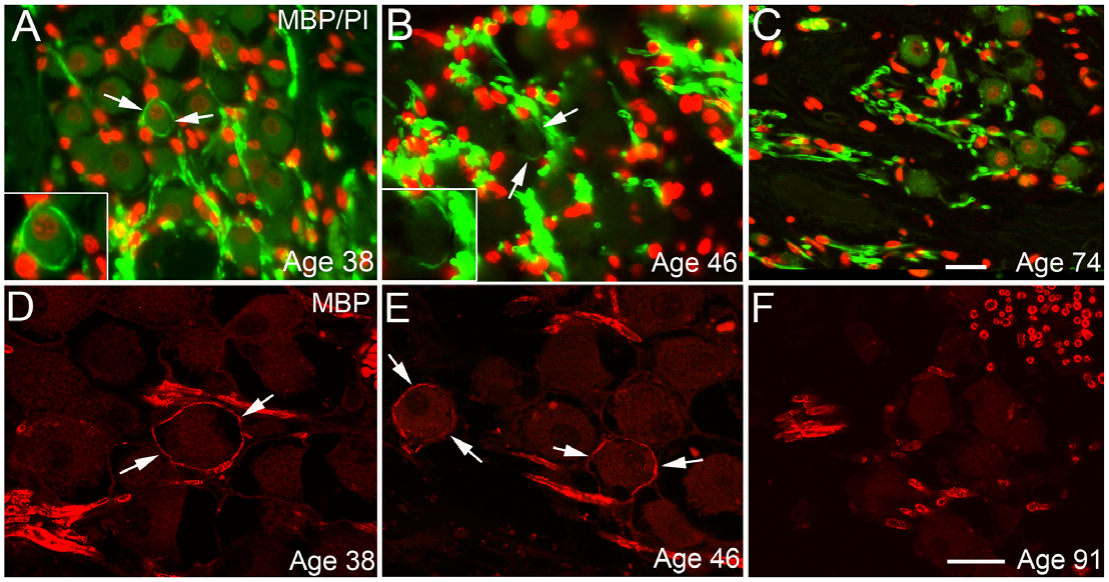
Age-related changes in MBP expression in human ears. **A–B:** A thin MBP^+^ myelin layer (arrows) around the perikarya of some type I neurons in both the middle (**A**) and basal (**B**) turns of ears from middle-aged donors. The inset in **A** shows a type I neuron with an intact MBP^+^ myelin layer at higher magnification. **C**: A MBP^+^ myelin layer was not observed enveloping any neurons in a mid-modiolar section taken from a 74-year-old donor. There was also a marked loss of MBP^+^ fibers between neurons and a reduction in the number of PI^+^ glial cell nuclei, relative to the middle-aged specimens. **D, E:** Confocal images of the MBP^+^ myelin layer (arrows) around the perikarya of some type I neurons in the spiral ganglion from a 38-year-old and a 46-year-old donor. **F:** A MBP^+^ myelin layer was not observed in a mid-modiolar section taken from a 91-year-old donor. Scale bar, 12 µm in **C** (applies to **A–C**); 12 µm in **F** (applies to **D–F**).

Unlike in mouse ears, the honey-comb-like staining pattern shown in Figs. 3 and 4, attributable to myelinated cell bodies, was not generally seen in the paraffin sections of human spiral ganglia we examined (Figs. 6A, E), with the exception of a small portion of the SNGs found in the middle-aged group (Figs. 7A, B, D, E). We counted 2510 SGNs in 48 mid-modiolar sections randomly selected from the middle-aged group (5–16 slides per ear) and found 82 of 2510 SGNs (3.26%) enclosed by a MBP^+^ myelin sheath. These neurons were evenly distributed across all three cochlear turns. We also examined 1707 SGNs from 32 mid-modiolar sections randomly selected from 6 aged human temporal bones. As shown in figures 7C and 7F, very few SGNs were enclosed by a MBP^+^ myelin sheath. Among the 1707 SGNs counted, only 7 neurons (less than 0.5%) had a thin MBP^+^ myelin sheath.

**Figure 8:**
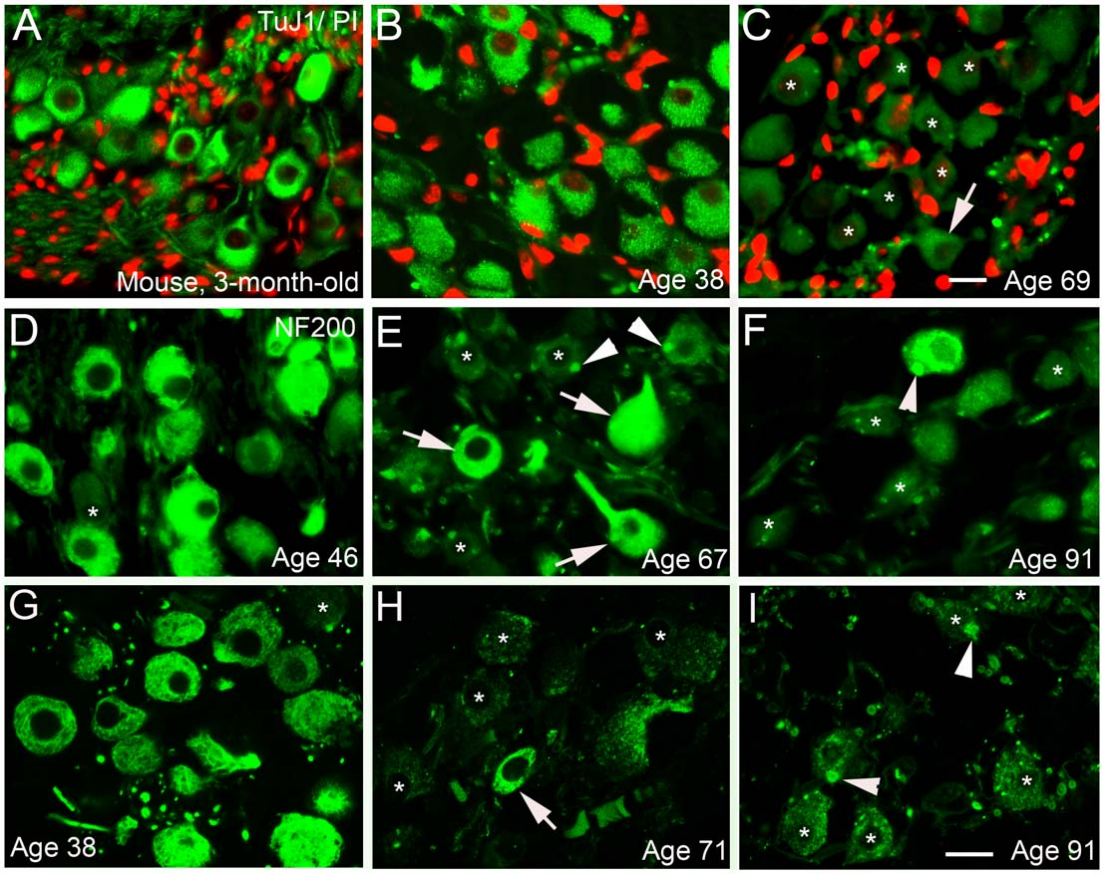
Age-related changes in TuJ1 and NF 200 expression in human ears. **A:** TuJ1^+^ neurons in a paraffin section from the middle turn of a young adult mouse. **B–C:** TuJ1^+^ neurons in the middle turns from (**B**) a 38-year-old donor (H18) and (**C**) a 69-year-old donor (H4). A marked decrease in TuJ1 immunostaining (asterisks) was seen in SGNs of the older ear. The white arrow indicates a neuron with a relatively normal level of TuJ1 immunoreactivity. Nuclei were counterstained with PI (red). **D, E, F:** A marked decrease of NF200 immunoreactivity was seen in many SGNs of older donors. NF200^+^ neurons (green) from the middle turns of (**D**) a 46-year-old (H26), (**E**) a 67-year-old (H19), and (**F**) a 91-year-old donor (H3). Asterisks indicate SGNs with a lower level of NF200 immunoreactivity. White arrows indicate SGNs with a level of NF200 immunoreactivity more similar to that of SGNs from the 46-year-old donor shown in **D**. Note the punctuate inclusions (white arrowheads) in the cytoplasm of some NF200^+^ SGNs indicating that neurofilament aggregation occurs in older ears. **G, H, I:** Confocal images showing a reduction in both the number of NF200^+^ neurons and decreased immunostaining intensity for NF200 in most surviving SGNs in older donors. Asterisks indicate SGNs with a lower level of NF200 immunoreactivity. A white arrow points to an SGN with a normal level of NF200 immunoreactivity. Punctuate inclusions (white arrowheads) were seen in the cytoplasm of many NF200^+^ SGNs in the 91-year-old donor. Scale bars, 8 µm in **C** (applies to **A–C**); 7 µm in **I** (applies to **D–I**).

Neurofilaments comprise the major intermediate filament system in mature neurons [54]. The class III β-tubulin isotype (TuJ1) is a microtubule component expressed exclusively in type I SGNs in ears from adult mice [55]. TuJ1immnoreactivity was examined in paraffin sections of both middle-aged and older human temporal bones. A marked decrease in immunostaining intensity for TuJ1 was seen in SGNs of the older group compared to the middle-aged group (Figs. 8B, C). Immunereactivity for another neuronal structural protein, neurofilament 200 (NF200), also was markedly diminished in the aged human spiral ganglion (Figs. 8D–I). In addition, NF200^+^ punctuate inclusions were seen in the cytoplasm of many SGNs in the older group, particularly within the basal turns (Figs. 8E, F, I).

**Figure 9:**
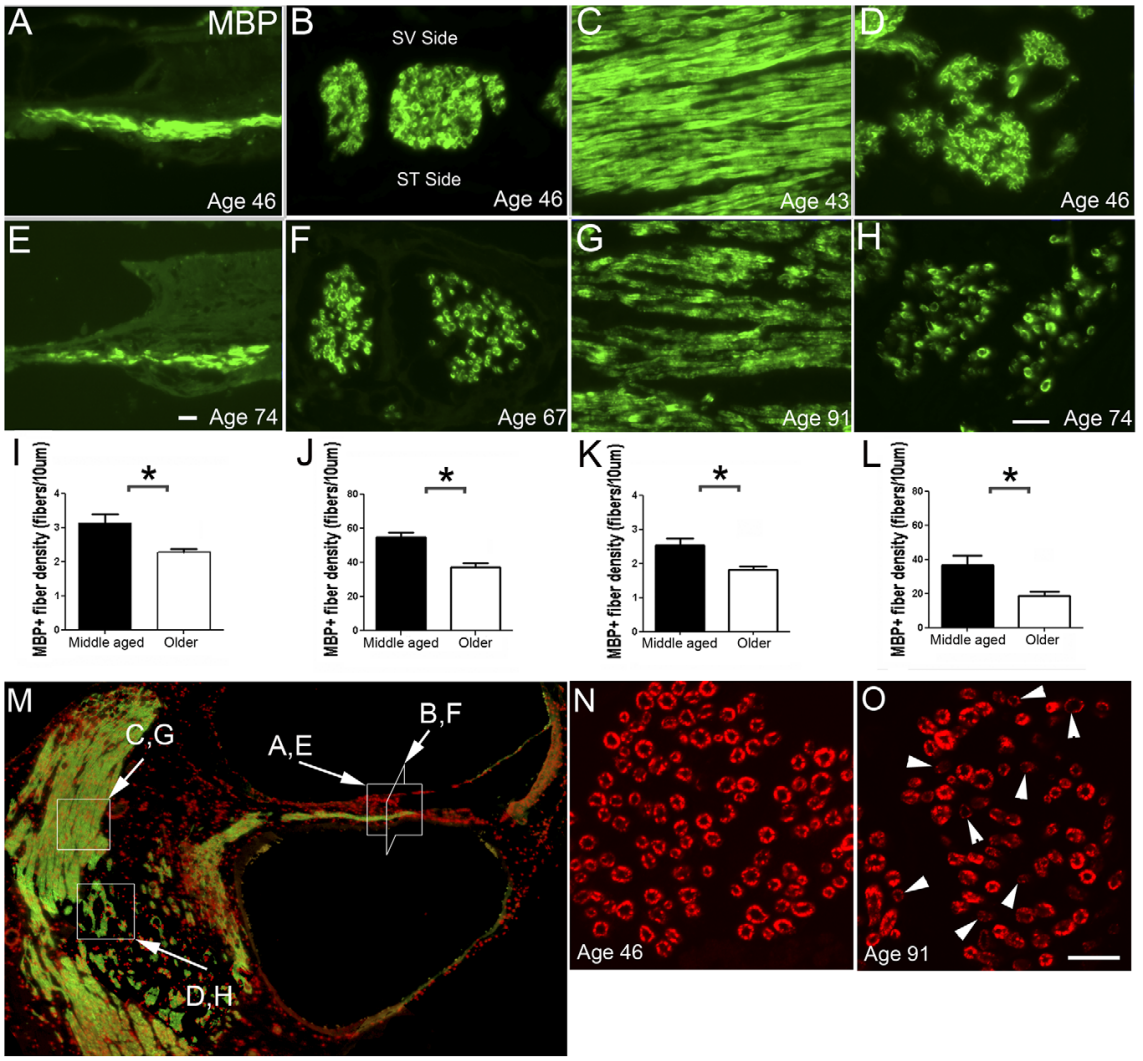
Age-related reduction of MBP^+^ fibers and immunostaining for MBP in human ears. **A–L; N–O:** A significant loss of MBP^+^ fibers occurred with age in both the peripheral and central portions of the auditory nerve. MBP^+^ nerve fibers are illustrated in peripheral (**A, B**) and central (**C, D**) portions of the auditory nerve in the ears taken from middle-aged (**A–D**) and old (**E–H**) donors. White boxes with associated letters in **M** illustrated the regions of the cochlea depicted in **A–H**. Counts of MBP^+^ fibers in both peripheral (**I, J**) and central (**K, L**) projections revealed a statistically significant reduction in fiber density in older compared to middle aged human cochleas using an unpaired t-test (* *p*<0.05). **N, O:** A reduction in the intensity of MBP immunoreactivity was seen in many myelinated fibers of a 91-year-old donor (white arrowheads). Scala vestibuli, SV; Scala tympani, ST. Scale bar, 10 µm in **E** (applies to **A, E**); 12 µm in **H** (applies to **B–D**; **F–H**); 12 µm in **O** (applies to **N, O**).

The number of MBP^+^ auditory nerve fibers within the osseous spiral lamina and the modiolus of the middle turn was determined in the middle aged and older ears (Figs. 9A–L). Figure 9M illustrates the regions of cochlea where the auditory nerve fibers were counted. Similar to the data shown for aged mice (Fig. 5), nerve fibers were counted in horizontal sections of the osseous spiral lamina (Figs. 9A, E). To validate the counting data obtained from horizontal sections, we also performed fiber counts in vertical sections of auditory nerve near the habenula perforata (Figs. 9B, F). Additionally, horizontal (Figs. 9C, G) and vertical (Figs. 9D, H) sections were evaluated to determine the fiber density in the central portion of the auditory nerve. The density of MBP^+^ fibers in both the peripheral (within the osseous spiral lamina) and central processes (within the modiolus) was significantly reduced in the older group as compared with the middle-aged ear (unpaired t-test, *p*<0.05).

Confocal microscopy revealed a reduced intensity of MBP immunostaining of many fibers in the central portion of the auditory nerve in older as compared to middle-aged ears (Figs. 9N, O). However, no significant difference was seen in the overall thickness of the MBP^+^ myelin sheath between the two groups. The thickness of MBP^+^ myelin sheath was measured in the osseous spiral lamina (peripheral processes) and modiolus (central processes) in the middle turn. The mean thickness of the MBP^+^ myelin sheath was 0.80±0.09 µm in the peripheral processes and 0.92±0.13 µm in the central processes in the middle-age group. Thicknesses were 0.75±0.08 µm in the peripheral processes and 1.02±0.22 µm in the central processes of the aged group. The difference in thickness with age was not significant (unpaired t-test, *p*>0.05).

## Discussion

CBA/CaJ mice have been widely used in studies of age-related functional and pathological alterations of the auditory system [43], [44], [46], [48], [56]–[59]. This mouse strain shows little change in peripheral auditory sensitivity until very late in life, e.g., significant ABR threshold shifts across a wide range of frequencies were not seen until over 2 years of age. In this study, we employed ABR wave I I/O functions to assess overall changes of auditory nerve function in CBA/CaJ mice. ABR wave I I/O function measurements allows examination of suprathreshold ABR amplitudes to assess the gross activity of the auditory nerve at high signal levels. The maximum wave I amplitudes and slopes of the I/O functions were reduced significantly in the aged mice at the tested frequencies, indicating a marked decline of auditory nerve activity. Similar age-related alterations in suprathreshold neural responses were previously reported in quiet-aged gerbils and older humans [8], [60]. The similar degenerative patterns of auditory nerve activity in older humans and aging CBA/CaJ mice led us to characterize and compare the cellular and molecular alterations in the spiral ganglia of aged CBA mice and older human temporal bone donors.

An important finding in this study was that age-related disorganization and degeneration of the myelin sheath occurs in the auditory nerve of the normal aging CBA/CaJ mice. Ultrastructural observations and MBP immunohistochemical analysis demonstrated that pathological alterations occur in both the myelin sheath and the cytoplasm of Schwann cells. Disorganization of myelin sheaths was common in degenerative SGNs but also was observed in relatively normal appearing SGNs in aging mice. Numerous dense inclusions were seen in the myelin-forming Schwann cells in the aged mice, but not in the young adult mice. The appearance of increasing numbers of dense inclusions has been described as a key age-related characteristic in the myelinating oligodendrocytes of aging monkeys [61]. It is likely that these dense inclusions in the myelin-forming glial cells are derived from the breakdown of the degenerating myelin sheaths.

In all complex nervous systems, neurons coexist with glial cells suggesting that neuron-glial interactions are a principal feature of neural function. Previous studies have demonstrated that Schwann cells not only myelinated axons and some neuronal cell bodies, but also play an import role in maintaining the long-term functional integrity and survival of neuronal cells (see review by Nave and Trapp 2008) [62], [63]. A large body of evidence supports the loss of intact myelin as underlying several neurological diseases, including multiple sclerosis, schizopherenia, inherited leukodystrophies of the CNS, and various peripheral neuropathies [64]–[66]. Thus, it is highly likely that the age-related loss of myelin sheath integrity and perhaps other Schwann cell support functions contribute to the degeneration of SGNs and declines in auditory nerve function with age.

Although no attempt was made to investigate the effect of hair cell loss on age-related SGN degeneration in this study, several studies have demonstrated that nerve degeneration can occur independent of hair cell loss [6]–[9], [67]. It remains unclear whether age-related myelin sheath loss is a primary event leading to secondary neuronal degeneration or vice versa. However, the presence of myelin abnormalities in neurons with an otherwise generally normal appearance suggests that structural alterations in the myelin sheath may be an early event in neuronal degeneration. A number of studies have demonstrated that integrity of the myelin sheath plays an important role in protecting nerve fibers from injury and maintaining the normal function of axons. For example, studies using spinal cord injury and multiple sclerosis models have demonstrated that intact myelin sheaths protect axons from glutamate excitotoxicity [68], [69] and limit the access of metalloproteinases released from inflammatory cells to the axons [70]. Other studies using mouse mutants carrying glia-specific defects have shown that glial cells play a critical role in regulating fast axonal transport as well as the functional integrity and long-term survival of axons [17], [63], [71], [72]. Hansen et al (2001) [73] demonstrated a reciprocal neurotrophic signaling process between SGNs and Schwann cells using an *in vitro* approach. Schwann cells genetically modified to secrete neurotrophins have been shown to enhance the survival of SGNs [74]. Moreover, a recent study showed that inactivation FGF receptor signaling in Schwann cells resulted in significant SGN loss in adult mice [75]. On the other hand, it is also likely that early biochemical alterations induced by ouabain in “generally normal appearing” neurons are not reflected by structural changes visualizable by electron microcopy. Additional studies using neurochemical approaches are needed to address this complex question. Multiple-experimental approaches, e.g., long-range live cell imaging of individual neurons and their processes, accompanied by the use of genetically manipulated models [76]–[78], will be helpful for identifying the early sub-cellular and molecular alterations leading to neuronal death in neurodegenerative diseases, including sensorineural hearing loss (see review by Laura et al [79]).

The progressive breakdown of myelin and degeneration of myelinated nerve fibers has been reported in normal aging and in age-related neurodegenerative diseases [32], [80], [81]. In non-human primates, myelin sheath abnormalities in cortical white matter correlate with age and decline of cognitive status [80]. Peters described common age-related myelin defects, including split myelin lamellae enclosing dense cytoplasmic collections, myelin balloons, the appearance of redundant myelin and the formation of circumferential splits in thick sheaths. In the spiral ganglion of aging CBA/CaJ mice, we observed split myelin lamellae both in SGNs exhibiting severe degenerative changes and in relatively normal appearing SGNs (Fig. 2). Numerous discontinuities accompanied the separation of myelin lamellae in most of the aged SGNs. Although splits in myelin sheath were seen in a few SGNs in young adult mice (Fig. 2B), no discontinuities were observed in young mice. The redundant myelin and split sheaths indicate continued myelin production [80]. These re-myelination features were not observed in aged mice, suggesting that re-myelination may not be a common event or may not occur at all in the aged spiral ganglion. We also measured the thickness of MBP^+^ myelin sheaths in the spiral ganglion and found no significant difference in thickness between young adult and aged mice or between middle-aged and older human donors.

Knowledge of myelin structure and the relationship between neurons, their processes, and Schwann cells in the spiral ganglion is primarily based on ultrastructural observations. The ultrastructure of the myelin sheath is very sensitive to the conditions of perfusion fixation, e.g., a shearing defect is often seen in the normal white matter due to its poor blood supply [27], [28]. It is technically challenging to study ultrastructural features of the myelin sheath in human auditory tissues for several reasons: 1) the inaccessibility of the auditory nerve to surgical biopsy; 2) the typically long postmortem fixation interval for human temporal bones; and 3) very limited access to well-fixed human ear tissues removed during surgical procedures. These facts, in addition to the small sampling areas, make ultrastructural studies of age-related histopathological changes in the human inner ear very difficult.

As reported here, the robust immunostaining of MBP in the human auditory nerve allows the rapid and efficient evaluation of age-related changes using a standard immunohistochemical assay on sections of formalin-fixed, paraffin embedded specimens. We have also established that immunostaining for MBP along with high-resolution confocal microscopy is an excellent alternative approach to electron microscopy for evaluating pathological changes of myelin sheaths in the auditory nerve. Immunohistochemical techniques have been used successfully to define the expression of several proteins in paraformaldehyde-fixed cryostat sections of human ears. These include basement membrane proteins [82], peripherin [83], synaptophysin [84], connexin [85], neurofilament [86], [87], aquaporins [88], a subclass of the ATP-binding cassette transporter proteins [89], tyrosine kinase receptor B and BDNF [90]. Using paraffin-embedded sections, Robertson et al [91] were the first to successfully demonstrate aggregation of cochlin expression in a human ear obtained from a DFNA9 donor. To our knowledge, no prior studies have defined age-related alterations of protein expression patterns in the human auditory nerve. Here, we employed archival, formalin-fixed and paraffin-embedded sections of human temporal bones collected from both middle aged and older donors. These temporal bone specimens were prepared using a microwave decalcification protocol previously established in our laboratory [92]. The data demonstrating that MBP antigenicity and cellular structural integrity can be well preserved using this accelerated decalcification protocol and paraffin embedment. These results also are consistent with our previous study demonstrating excellent immunostaining for several ion transport proteins on similarly processed human temporal bone specimens [93].

Another interesting finding was the apparent loss of the already limited MBP^+^ myelin sheath surrounding the perikaryon of a small percentage of SGNs in older human donors. Unlike in mice, the somata of most human SGNs are unmyelinated [18]–[22]. An ultrastructural study on a temporal bone from a 21-year-old normal donor found that only 4% (16 out of 356) of large neurons and 2% (1 out of 52) of small neurons were myelinated [20]. Arnold [19] reported that about 2% of SGNs in two middle aged donors (aged 61 and 63 years) had a thin myelin sheath. Another study in a series of human donors aged 65–92 years documented that approximately 5.8% of SGNs were myelinated [18]. In this study, we found that about 3.3% of SGNs (82 out of 2510) in the middle-aged group (38–46) were enveloped by a MBP^+^ myelin sheath. In contrast, in the older group, only about 0.46% of SGNs (7 out of 1707) were surrounding by MBP^+^ myelin, strongly suggesting either the loss with age of the myelin sheath in most myelinated neurons or the loss of the neurons themselves. However, this assumption is not supported by the observation from a 75-year-old donor reported by Ota and Kimura [18], although a relatively small number of neurons were counted (11 out of 39) and only a portion of Rosenthal's canal was sampled. The functional significance of the small number of myelinated type I SGNs in the human ear remains unknown. This differential feature between human and most other mammalian species may reflect the need for slower and more precise neural conduction in the human ear [21], [22].

Age-related changes of MBP gene and protein expression have been reported in the rat peripheral nervous system including the sciatic nerve and spinal root [94], [95]. Wang et al [96] documented that MBP immunoreactivity is significantly lower in the white matter of subjects with Alzheimer disease than in subjects without cognitive impairment, suggesting that a deficiency of MBP expression in white matter may contribute to an age-associated decline in cognition. The data here demonstrate a significant reduction of MBP^+^ fibers in both the peripheral and central processes of the auditory nerve and the loss of MBP^+^ myelin sheaths in type I SGNs of both aging CBA/CaJ mice and older humans. A decline of auditory nerve function was also associated with changes in MBP expression in normal aging mice. In addition to its importance in myelin formation, MBP has been shown to interact with a number of polyanionic proteins including tubulin [97], actin [98], tropmyosin [99], Ca^2+^- calmodulin [100]–[102] and clathrin [103]. This suggests that MBPs may be involved with the transmission of extracellular signals to the cytoskeleton in Schwann cells. Further molecular and functional studies of this very abundant protein are needed to better understand the causes of myelin loss and axon degeneration in neurodegenerative disorders including age-related hearing loss.

## Materials and Methods

### Animals

All aspects of the animal research were conducted in accordance with guidelines of the Institutional Animal Care and Use Committee (IACUC) of the Medical University of South Carolina (MUSC). Experimental procedures were reviewed and approved by the IACUC at MUSC under protocol number #AR2290. Adult CBA/CaJ mice were bred in-house in a low-noise environment in the MUSC Animal Research Facility with original breeding pairs purchased from The Jackson Laboratory (Bar Harbor, ME). All mice received food and water *ad libitum* and were maintained on a 12-hour light/dark cycle. Mice of both genders aged 1–3 months (young adult group) and 23–28 months (aged group) were used in the study. Our preliminary results showed no significant difference in the morphological or immunohistochemical characteristics of the inner ear between 1 and 3 month-old mice. Throughout this paper, the term “young adult mice” applies to 1–3-month-old mice and “aged mice” applied to 23–28-month-old mice. Prior to data acquisition, mice were examined for signs of external ear canal and middle ear obstruction. Mice with any symptoms of ear infection were excluded from the study. A total of 17 young adult and 14 aged mice were used in this study.

### Physiological procedures

Mice were anesthetized by intraperitoneal injection of xylazine (20 mg/kg) and ketamine (100 mg/kg) and placed in a head holder in a sound-isolation room. Auditory brainstem responses (ABRs) were recorded via customized needle electrodes inserted at the vertex (+) and test-side mastoid (−), with a ground in the control-side leg. The acoustic stimuli were generated using Tucker Davis Technologies equipment III (Tucker-Davis Technologies, Gainesville, FL, USA) and a SigGen software package. The calibration was completed using a Knowles microphone in a probe tube clipped to the pinna. The signals were delivered into the mouse ear canal through a 10 mm long (3–5 mm diameter) plastic tube. ABR thresholds, defined as the lowest sound levels at which the response peaks are clearly present as read by the eye from stacked wave forms, were obtained. ABRs were evoked at half octave frequencies from 4 to 45 kHz with 5 ms duration tone pips with cos^2^ rise/fall times of 0.5 ms delivered at 31/s. Sound levels were reduced in 5-dB steps from 90 dB SPL to 10 dB SPL. For ABR amplitudes vs. level functions (I/O function), the wave I peaks were identified by visual inspection at each sound level with the peak-to-peak wave I amplitude computed. At each sound level, 300–500 responses were averaged, using an “artifact reject” protocol whereby response waves were discarded when peak-to-peak amplitudes exceeded 50 mV. Physiological results were analyzed at individual frequencies from 4.0 to 40 kHz.

### Human ear tissue preparations

As shown in Table 1, the temporal bones used in this study were collected from ten human subjects including 4 donors aged 38–46 years (two female and two male; average = 43.3 years; middle-aged group) and 6 donors aged 63–91 years (two female and four male; average = 71.2 years; older group). The temporal bones were selected from a collection of human temporal bones obtained as part of a longitudinal study of age-related hearing loss conducted by the Hearing Research Program at the Medical University of South Carolina (MUSC). These donors, especially those over 60 years of age, were mainly recruited from patients in the MUSC Hospital System. Procedures for the collection and use of the temporal bones were approved by the MUSC Institutional Review Board under protocol HR# E-607R, with written consent obtained in all cases.

At autopsy, brain tissues including the brainstem and major blood vessels were carefully elevated from the cranial cavity. All cranial nerves were severed with scissors or a long-handled scalpel. The auditory nerve and internal auditory arteries were sectioned outside the internal auditory canal to avoid an avulsion of the nerve from the temporal bone. The temporal bones were removed using an oscillating 38-mm trephine saw according to previously described techniques [2], [104]. Immediately after removal, the temporal bones were fixed by perilymphatic perfusion with 2 ml of a 10% neutral buffered formalin solution. The pre-fixation process included elevation of the tympanic membrane, removal of the stapes and perforation of the round window membrane. The fixative was perfused gently through the oval window using a blunt-tip, 16-gauge needle covered with an appropriately-sized tygon tubing. The perfused temporal bones were then immersed in fixative for 12–22 hours followed by rinsing and decalcification using a microwave protocol as per our previous description [92]. The total time of decalcification was between 3–6 weeks. During the process of decalcification, temporal bones were gradually trimmed using roungeurs and a No. 15 blade scalpel to remove most of the hard bone encasing the cochlear and vestibular apparatus. The decalcified inner ears were dehydrated and embedded in paraffin (Paraplast, XTRA, Oxford Labware, Sherwood Medical, St. Louis, MO). Serial sections were cut at a thickness of 4 µm and mounted two per slide on Colorfrost®Plus slides (Fisher Scientific, Pittsburgh, PA). Every 25^th^ section was stained with H&E for the evaluation of the morphological preservation. The sections were further processed with immunohistochemical labeling for MBP, neurofilament 200, and class III β-tubulin (TuJ1) as described below.

### Transmission electron microscopy and immunohistochemical analysis

Following ABR measurements, young adult and aged CBA/CaJ mice were processed for either ultrastructural or immunohistochemical analysis. For ultrastructural observations, the anesthetized mice, including three 3-month-old and four 26–28-month-old CBA/CaJ animals, were processed as described previously [105], [106]. The animals were perfused via a cardiac catheter, first with 10 ml of normal saline containing 0.1% sodium nitrite, and then 15 ml of a mixture of 4% paraformaldehyde and 2% glutaraldehyde in 0.1 M phosphate buffer, pH 7.4. After removing the stapes and opening the oval and round windows, 0.5 ml of fixative was perfused gently into the scala vestibuli through the oval window. The inner ears were dissected free and immersed in fixative overnight at 4°C. Decalcification was completed by immersion in about 50 ml of 120 mM ethylenediamine tetracetic acid (EDTA), pH 7.2, with gentle stirring at room temperature for 2–3 days with daily changes of the EDTA solution. The tissues were postfixed with a 1% osmium tetroxide for 1 hour, dehydrated, and embedded in Epon LX 112 resin. Semi-thin sections approximately 1 µm thick were cut and stained with toluidine blue. Ultra-thin sections were stained with uranyl acetate and lead citrate and examined by electron microscopy. All three turns of the inner ears taken from the four aged and three young control mice were examined in this study. Eight to eleven sections per turn in each mouse were examined by electron microscopy. The eighth nerves of aged and young adult mice were examined from synapses with hair cells to the main nerve trunk within the internal auditory canal. Measurements of the thickness of the compact myelin sheath in young adult mouse ears (n = 3) were performed on electron micrographs. Measurements were made on 41 SGNs and 81 auditory nerve fibers within Rosenthal's canal selected randomly from sections of all three turns. Similar measurements of the thickness of the compact myelin sheath in aged mouse ears (n = 4) were made on 49 SGNs and 44 auditory nerve fibers within Rosenthal's canal selected randomly from the sections of all three turns.

For immunohistochemistry, the mouse inner ears were prepared following the procedure described above but substituting 4% paraformaldehyde as fixative, decalcified with EDTA, cryoprotected in 30% sucrose in PBS and embedded in Tissue-Tek OCT compound (Electron Microscopy Science, FT. Washington, PA). The sections were pretreated with ice cold acetone and methonal for 3 and 7 min, respectively, followed by 0.1% TritonX-100 for 10 min. Then, the pretreated sections were blocked with 0.1% BSA and 0.3% TritonX-100 in PBS for 45 min, followed by incubation with a primary antibody at 4°C overnight.

For immunostaining on human temporal bones, deparaffinized and rehydrated sections were pretreated with an antigen retrieval protocol as previously described [100]. The pretreated sections of human ear tissue were immersed in blocking solution as described above and incubated with a primary antibody at 4°C for 12–24 hours. As shown in Table 2, the primary antibodies used in this study were mouse anti-myelin basic protein (MBP, 1∶250,SMI-94; Abcam, Cambridge, MA), rabbit anti-class III β-tubulin (TuJ1, 1∶200, MRB-435p; Covance, Emeryville, CA), and mouse anti-neurofilament 200 (NF 200, 1∶200, Clone N52, N0142; Sigma, Atlanta, GA). Biotinylated secondary antibody binding was detected with FITC-conjugated avidin D (1∶150) (Vector, Burlingame, CA) or Texas-red conjugated avidin D (1∶150) (Vector, Burlingame, CA) for visualization. Nuclei were counterstained with bisbenzimide or propidium iodide (PI). Staining of control sections for all of the primary antibodies included omission of the primary antibody or substitution with similar dilutions of non-immune serum of the appropriate species. No regionally specific staining was detected in any of these control experiments.

**Table 2:** Antibody Characterization.

Antibody	Immunogen	Species	Catalog #	Supplier	Dilution
			Lot #		
MBP	TADPKNAWAQD AHPADPGSRP, corresponding to amino acids 70–89 of human myelin basic protein	Mouse	Ab24567	Abcam	1∶250
Neurofilament 200	200 kDa neurofilaments in rat spinal cord extract	Mouse	N0142 Clone N52	Sigma	1∶200
Class III *β*-tubulin (TuJ1)	Microtubules derived from rat brain	Rabbit	SC-68377	Santa Cruz	1∶200

The mouse monoclonal antibody (clone SMI-94) to myelin basic protein reacts with a recombinant fragment of human myelin basic protein corresponding to amino acids 70–89 (manufacturer's technical information). This antibody detects myelin in a wide range of mammalian species including rat, mouse, rabbit, dog, monkey and human. We processed immunoblots on young adult mouse brain and auditory nerve extracts. An intensely stained band was seen around 21 kDa molecular weight (data not shown). The mouse monoclonal antibody to neurofilament 200 recognizes the neurofilament of molecular weight 200 kDa in rat spinal cord extracts (manufacturer's technical information). When tested by immunoblotting on pig neurofilament polypeptides, the antibody reacts with an epitope in the tail domain of neurofilament 200, also referred to as the H-subunit, which is present on both the phosphorylated and non-phosphorylated forms of this polypeptide. The staining patterns with neurofilament 200 seen here in the mouse and human auditory nerve were similar to those in previous descriptions [87], [107]. The neuronal class III β-tubulin (TUJ1) rabbit monoclonal antibody was raised against microtubules derived from rat brain and is highly reactive to mammalian neuron-specific class III β-tubulin, but not to glial β-tubulin (manufacturer's technical information). A previous study demonstrated that TuJ1 was highly expressed in type I SGNs of the young adult mouse [50].

Quantitative analysis of MBP^+^ fibers was performed using four ears from the young adult mouse group, five ears from the aged mouse group, four ears from the middle-age human group, and six ears from the aged human group. Nerve fiber count data were collected from five to six 15 µm mid-modiolar frozen sections per mouse ear and ten to sixteen 4 µm mid-modiolar paraffin sections per human ear. Nerve fiber counts in the osseous spiral lamina regions were performed within boundaries between a distal site near the habenular opening and a proximal site near the spiral ganglia and between the scala tympani and scala vestibuli margins. Measurements on horizontal sections of the nerve fibers were made in areas about 20–30 µm from the habenular opening in mouse ears (Figs. 5A, B) and about 50–70 µm from the habenular opening in human ears (Figs. 9A, E). For human ears, nerve fiber counts were performed just before their exit from the habenula perforata. This area ranged from 1160 to 2300 µm^2^ on vertical sections of the auditory nerve (Figs. 9B, F).

Values for the sampling area and numbers and thickness of MBP^+^ fibers were determined and collected using the Automatic Measurement Feature of AxioVison 4.8 software (Carl Zeiss Inc., Jena, Germany). The sections used for counting were at least 30 µm apart for mouse tissue analysis (fiber counting was performed on 1 in every 3 serial sections) and at least 40 µm apart for human tissue analysis (fiber counting was performed on 1 in every 10 serial sections). Confirmation of MBP labeling was performed by focusing through the full height of the section. Note that throughout the paper, the term “auditory nerve” refers to the peripheral part of the VIII nerve extending from synapses with hair cells in the organ of Corti to the main nerve trunk within the modilous and extending into the internal auditory canal.

The sections were examined with a Zeiss Axio Observer (Carl Zeiss Inc., Jena, Germany), and the captured images were processed using Image Pro Plus software (Media Cybernetics, MD) and AxioVison 4.8 (Carl Zeiss Inc., Jena, Germany). Adobe Photoshop CS2 was employed to adjust brightness, contrast, and sharpness of images with identical settings for all panels. Alterations were not performed on images used for quantitative purposes.

### Data analysis

Unless otherwise specified, all data in the figures are presented as mean ± standard error of the mean (SEM). Data for the ABR thresholds, ABR wave I amplitude I/O functions, and the densities and thicknesses of MBP^+^ fiber were analyzed by two tailed, unpaired *t* test (SPSS, Chicago, IL). A value of *p*<0.05 was considered to be statistically significant.
